# Environmental Factors and Seasonality Affect the Concentration of Rotundone in *Vitis vinifera* L. cv. Shiraz Wine

**DOI:** 10.1371/journal.pone.0133137

**Published:** 2015-07-15

**Authors:** Pangzhen Zhang, Kate Howell, Mark Krstic, Markus Herderich, Edward William R. Barlow, Sigfredo Fuentes

**Affiliations:** 1 Department of Agriculture & Food Systems, The University of Melbourne, Parkville, Vic, Australia; 2 Australian Wine Research Institute, Port Melbourne, Vic, Australia; 3 Australian Wine Research Institute, Urrbrae, SA, Australia; Fresno, UNITED STATES

## Abstract

Rotundone is a sesquiterpene that gives grapes and wine a desirable ‘peppery’ aroma. Previous research has reported that growing grapevines in a cool climate is an important factor that drives rotundone accumulation in grape berries and wine. This study used historical data sets to investigate which weather parameters are mostly influencing rotundone concentration in grape berries and wine. For this purpose, wines produced from 15 vintages from the same Shiraz vineyard (The Old Block, Mount Langi Ghiran, Victoria, Australia) were analysed for rotundone concentration and compared to comprehensive weather data and minimal temperature information. Degree hours were obtained by interpolating available temperature information from the vineyard site using a simple piecewise cubic hermite interpolating polynomial method (PCHIP). Results showed that the highest concentrations of rotundone were consistently found in wines from cool and wet seasons. The Principal Component Analysis (PCA) showed that the concentration of rotundone in wine was negatively correlated with daily solar exposure and grape bunch zone temperature, and positively correlated with vineyard water balance. Finally, models were constructed based on the Gompertz function to describe the dynamics of rotundone concentration in berries through the ripening process according to phenological and thermal times. This characterisation is an important step forward to potentially predict the final quality of the resultant wines based on the evolution of specific compounds in berries according to critical environmental and micrometeorological variables. The modelling techniques described in this paper were able to describe the behaviour of rotundone concentration based on seasonal weather conditions and grapevine phenological stages, and could be potentially used to predict the final rotundone concentration early in future growing seasons. This could enable the adoption of precision irrigation and canopy management strategies to effectively mitigate adverse impacts related to climate change and microclimatic variability, such as heat waves, within a vineyard on wine quality.

## Introduction

Predicting the quality of wine by analysing the vineyard weather parameters has proven to be an attractive, yet elusive goal for scientists and viticulturists. Mean January temperature (MJT) and growing degree days (GDD) are the most commonly used viticultural weather parameters to estimate the quality and potential price points of the wine for *Vitis vinifera* L. cultivars [1]. More broadly, the number of days with a specific range of temperatures, solar radiation and relative humidity during grape ripening has been associated with resulted wine quality in several Australian wine regions [2]. Vineyard microclimatic data, including visible light radiation and canopy temperature were also shown to be good indicators of the final quality for Sauvignon blanc wine in South Africa [3].

Newly available databases provide more comprehensive environmental information to viticulturists that could be helpful to select agroclimatic regions suitable for winegrowing. Specifically, the Australian water availability project (AWAP) has established an Australian-wide fine scale climate map database, which can provide precise historical weather data (from 1900 onwards) for any Australian agricultural location [4]. This gives an opportunity to study the influence of climatic and microclimatic parameters on grape and wine quality throughout historical seasons. Thus, wine quality estimation models could be established to characterise and estimate quality trait parameters for wine based on the AWAP dataset for a specific viticultural site or region in Australia.

Rotundone is an oxygenated bicyclic sesquiterpene that contributes to the ‘black pepper’ character of Shiraz grapes and wine, which is favourable to many wine consumers, especially experienced wine drinkers [5–7]. This peppery characteristic is stylistically crucial to high quality Australian Shiraz wine [8]. Unlike many other wine aroma compounds, rotundone originates from the grapes berry exocarp (skin) [9], and is extracted during the wine primary fermentation process [8]. Rotundone is a very stable chemical compound in the wine matrix, with little loss under different bottling and accelerated aging conditions [10]. Therefore, grape rotundone concentration is crucial to the ‘peppery’ character in the finished wine, and the rotundone found in wine can be used as an accurate proxy for the amount found in wine grapes from the same vintage. And as rotundone concentration is directly related to the sensory quality of the Shiraz wine, measuring this compound and its relation to environmental/weather measures provides an opportunity to predict and model wine quality.

The influence of vineyard microclimate on the production of rotundone is currently elusive in the literature. In general terms, Shiraz vines grown in cooler climates have been associated to higher rotundone concentration in grape berries and therefore in wines produced. The latter, based on the organoleptic observation that the most ‘peppery’ Shiraz wines originates from ‘cooler’ geographic production areas [8]. Most recently, it has been demonstrated that rotundone in berries varies considerably within the same vineyard [11], within the same vine and even within the same bunch [12], which may be related to the differences in bunch zone microclimate. This same research showed that temperatures at the grape surface, bunch zone and ambient air, were important for rotundone production, with temperatures exceeding 25°C been associated to negative impacts on berry rotundone concentration.

Seasonal water balance in a vineyard is critical to the development of many flavour and quality traits in grapes and wine [13, 14]. Vine water deficit has been linked to: i) increments of monoterpenoids and isoprenoids concentration in resulted Merlot wine [14]; ii) affecting the phenylpropanoid, abscisic acid, isoprenoid, carotenoid, amino acid and fatty acid metabolic pathway in Cabernet Sauvignon and Chardonnay [13]; iii) significantly increasing the transcription of one sesquiterpene related terpenoid synthetase at maturity in Chardonnay and at early ripening stages in Cabernet Sauvignon [13]. Even though, the metabolic pathway of rotundone has not been fully unravelled, one possible precursor namely α-guaiene has been reported [15], and there is the possibility that water deficit may affect the rotundone biosynthetic pathway. For example, a recent study showed that increased irrigation over the veraison-harvest period resulted in higher rotundone concentration in grapes and resulted wine [16].

Solar exposure of grape berries is also important for the accumulation of berry aroma and flavour compounds. It has been shown that moderate solar exposure increased grape berry monoterpenes concentration at harvest in Traminette grapes [17]. On the contrary, complete sunlight exclusion from berries, especially from veraison to harvest, significantly inhibits the synthesis and accumulation of monoterpenes in Muscat grapes [18]. Sunlight exposure influences grape metabolites synthesis either by increasing grape surface temperature or via higher UV-B radiation [12, 17, 19]. The latter also influences the concentration of abscisic acid, salicylic acid, jasmonic acid and ethylene in plants, which play important roles in the up-regulation and down-regulation of gene expression for plant immune responses, especially terpenoids synthesis [19–21]. The influence of sunlight on the concentration of rotundone has not yet been studied. Nevertheless, previous studies suggest that sunlight could be a factor important to the final concentration of rotundone in grapes at harvest.

The influence of weather parameter on grape quality varies at different grapevine physiological stages, especially when some compounds such as sesquiterpenes mainly develop at certain time of the ripening stage in *Vitis vinifera* cultivars [22]. Bicyclic sesquiterpenes were mainly detected in the post-veraison period of berry development in Shiraz [21], and therefore the weather parameters during this period may be more important to the concentration of rotundone in grapes at harvest. In western Victoria (Australia), grapevines start the annual cycle around late September to October, where the dormant vines enter the bud burst stage and start to grow. In this region, veraison (start of berry softening and colour change) occurs typically from January to early February for the Shiraz cultivar [23], but usually occurs in middle February for the studied vineyard. Commercial harvest typically happens from middle March to early April in this region, but usually middle to late April for the studied vineyard. The critical stage for quality grape production is from the post-veraison to harvest period, where grape berries rapidly accumulate sugar, change colour, shift metabolism and accumulate flavour compounds [24]. It is likely that meso- and micro-climatic parameters at the post-veraison period play a critical role in the final rotundone concentration in wine grapes, as rapid rotundone accumulation has been only observed to occur around two weeks before harvest [24]

This paper describes a detailed study on the interaction between weather parameters and rotundone concentration in Shiraz grapes and wine over 15 growing seasons (from 1996–97 until 2013–14). This study implements interpolation techniques to obtain hourly air temperature in the bunch zone from historical growing seasons, and establishes descriptive models to characterise grape and wine rotundone concentrations in berries within a season. Even though the models described in this paper are site specific, the modelling techniques are applicable to any vineyard with adequate environmental and berry quality records and information. These techniques can be used to link historical weather data with rotundone production considering the availability of critical parameters, and therefore potentially be applied to predict rotundone concentration at harvest from real time weather information in the growing season.

## Materials and Methods

### Site and plant material description

All grapes and wines used for this study were obtained from a commercial vineyard planted with *Vitis vinifera* L. cv Shiraz (The Old Block, Mount Langi Ghiran 37.31°S, 143.15°E) located in the Grampians wine region of Victoria, Australia. The vineyard was planted in 1968 on its own roots at 3.0 m between rows and 1.8 m between vines, with rows oriented northeast to southwest. Grapevines are trained to a vertical shooting positioning trellis (VSP). Regional climatic data is described in the following sections.

### Ethics statement

All the samples in this study were collected from private land (Mount Langi Ghiran), and the owners of the vineyards gave permission to conduct the study on these sites. No specific permissions were required for these locations, because there were no endangered or protected species in these areas, and this study did not involve endangered or protected species.

### Wine and grape sampling for rotundone concentration assessment

Shiraz wines from 15 different vintages were studied, with the earliest growing season from 1995–1996 (vintage 1996) and the most recent growing season of 2013–2014 (vintage 2014). Due to the limitation of wine stock from historical seasons, only selected seasons were studied. Two bottles of Shiraz wine produced in each selected vintage were sampled using 100 ml sealed bottles, and transferred to the laboratory for chemical analysis. It has been shown that winemaking techniques potentially affect the concentration of rotundone in wines as rotundone accumulates during the first few days of primary fermentation and remains constant afterward [9]. However, the winemaking protocol at the studied commercial winery was consistent throughout the studied vintages. Therefore, the winemaking process was not considered as a major factor contributing to the differences in wine rotundone concentration in this study.

Grape bunches were randomly sampled across the vineyard in triplicate (2kg per field replicate) at fortnightly intervals from 80% veraison to commercial harvest in three continuous seasons (2011–12, 2012–13 and 2013–14). Grape samples were collected in zip-lock plastic bags, frozen at -20°C and transferred to the laboratory in styrofoam boxes on dry ice, and stored at -20°C before chemical analysis. The rotundone concentration in grapes and wine has previously been reported to be stable under proper storage conditions, and unlikely to change drastically during wine aging [10].

### Preparation of samples and SPME-GC-MS analysis of rotundone

Grape and wine samples were prepared for rotundone analysis based on the protocol described by Siebert et al. [5]. The 100 ml of wine samples described before were sub-sampled before analysis. 100μL of d5-rotundone (516ng/ml in ethanol) was added as internal standard to each sample, and then subjected to solid phase extraction (SPE) performed as reported previously [5]. For each of the grape samples, 100 g of destemmed grapes were sub-sampled before being homogenised using a hand-held blender. Sub-samples were centrifuged to separate the juice and solid parts. The solid parts were mixed with 30 ml of ethanol, 30 ml of water and 100μL of d5-rotundone (516ng/ml in ethanol) as internal standard, then shaken for 24 hrs at 22°C and sonicated before adding the juice back. Sub-samples were then centrifuged and filtrated (1.6 μm glass fibre) to obtain berry extract filtrate, which was topped up to 200ml with MilliQ deionised water before subjected to solid phase extraction (SPE) following the same method used for wine samples. For both grape and wine samples, the SPE residue supernatant collected was air dried with nitrogen, and reconstituted in 0.5 ml of ethanol and 9ml of MiliQ deionised water. The samples were then analysed and quantified with SPME-GC-MS using the parameters as described by Geffroy et al. [24].

### Regional historical climatic data and irrigation scheduling

The weather data of each selected season was obtained from the nearest Bureau of Meteorology (BOM) weather station located at Ararat Prison (Australian BOMƒ Station No. 089085), which is approximately 15.5 km west from the vineyard. The long-term mean January temperature (MJT) recorded at this weather station was 18.9°C with annual average rainfall of 588 mm, which classify it as a cool climate wine region [23]. The seasonal MJT and mean daily solar exposure (E_s_) from October to harvest were also calculated for each studied season (S1 Table). The vineyard is under drip irrigation system with dripper spacing of 0.5 m, and discharge of 1.5 L h^-1^ since 1998. Before 1998, a different drip irrigation layout was in place with dripper spacing of 1.8 m, and dripper output of 4 L h^-1^. Irrigation data from each vintage was recorded by the winery. Crop evapotranspiration (ET_c_) from each season has been calculated as reference evapotranspiration (ET_o_) × crop coefficient (K_c_), where ET_o_ was calculated from the temperature and dew point data (BOM station) based on the simplified Penman formula [25], while K_c_ used were 0.28, 0.35, 0.42, 0.43, 0.15 and 0 for October, November, December-January, February-March, April and May, respectively, based on Pudney et al. [26]. Cumulative growing degree-days (DD_s_) from each season from Oct to harvest were calculated from the BOM station temperature data with a base temperature of 10°C following the method described by Gladstones [22] and a proposed interpolation method to obtain half-hourly temperature data from daily maximum and minimum temperatures, which will be described in detail later in this paper. The MJT, E_s_, irrigation volume, ET_c_ and DD_s_ for studied seasons are described in S1 Table.

### Interpolated vineyard weather data to obtain thermal time

A simulated temperature model (STM) obtained with minimal data, and specific for the experimental vineyard, was developed based on the AWAP database. The Australian Bureau of Meteorology have generated high-resolution spatial climate maps for AWAP based on the Australian Data Archive for Meteorology [4], which includes daily/monthly rainfall, temperature and solar exposure data from 1900 to 2014 (continuously updated). Weather data of the studied vineyard was extracted using the vineyard GPS location from the AWAP high-resolution spatial climate maps using ArcMap software (ver.10, Esri. Redlands, CA, USA). Mean daily maximum temperature (T_max_), mean daily minimum temperature (T_min_) and mean daily solar exposure (E_vh_) from veraison to harvest were extracted for each studied season. Since historical veraison time from the studied vineyard was not available, the estimated veraison time was the 15^th^ February for most of the growing seasons (Mr Damien Sheehan, vineyard manager, personal communication: dsheehan@langi.com.au). However, for the growing seasons where harvest was conducted earlier than the 14^th^ April, the estimated veraison time was approximately 60 days before harvest (Mr Damien Sheehan, dsheehan@langi.com.au). Water balance (P_wb_) for each growing season (October to harvest) was calculated using a simplified water balance method as total rainfall + total irrigation − ET_c_, where total rainfall was calculated from AWAP maps. Furthermore, T_max_, T_min_, E_vh_, total rainfall, P_wb_ and wine rotundone concentration for studied seasons are described in S2 Table.

The STM estimates the half-hourly temperatures of the vineyard from veraison to harvest based on the daily maximum and minimum temperature obtained from AWAP high-resolution spatial climate maps using the piecewise cubic hermite interpolating polynomial (PCHIP) function and a customised code written in MatLab ver. 2014a (The MathWorks, Inc. Matick. MA. USA). The PCHIP function can be expressed as:

$${\text{T}}_{\text{i}}=\text{PCHIP}\left({\text{t}}_{\text{m}},{\text{ T}}_{\text{m}},{\text{ t}}_{\text{i}}\right),$$

where T_i_ is the predicted temperature at the time point t_i_, T_m_ is the daily maximum/minimum temperature at their corresponding time t_m_. PCHIP finds temperature values of an underlying interpolating function T(t) at each time point between the time points of daily maximum and minimum temperatures, such that: on each time subinterval, t_k_ ≤ t ≤ t_k+1_, T(t) is the cubic Hermite interpolant to the given temperature values and certain slopes at two endpoints; T(t) interpolate T_m_, for example, T(t_mj_) = T_mj_, and the slopes at the t_mj_ are chosen in a way that T(t) preserves the shape of the (t_m_, T_m_) data and respects monotonicity. This means that, on intervals where the data are monotonic, so is T(t); at points where the data has a local extrema, so does T(t).

**Fig 1:**
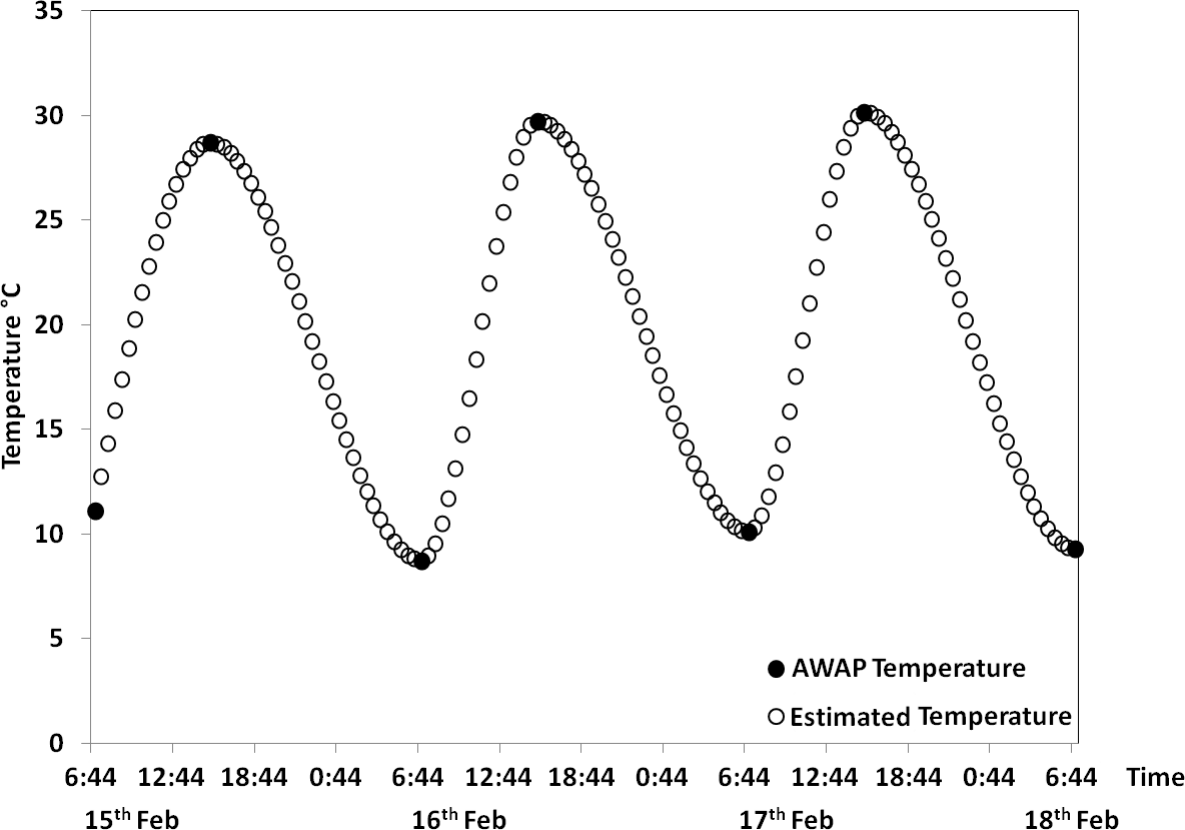
Half-hourly temperature data (clear circles) estimated from AWAP daily maximum/minimum temperature data (black-filled circles) using the simulated temperature model (STM) for selected days from the 1995–96 growing season.

Temperatures from veraison to harvest were estimated at half-hourly intervals using the STM proposed for all growing seasons studied. The AWAP temperature data and predicted half-hourly temperatures from STM were plotted against time (Fig 1). Vineyard thermal time was calculated as degree hours using the data obtained from STM following the established protocol [27], which represents the heat-hours accumulated in the vineyard (S3 Table). Percentage of degree hours above 35°C (DH_35_), above 30°C (DH_30_) and above 25°C (DH_25_) from the total degree hours were calculated using a customised code written in MatLab ver. 2014a (The MathWorks, Inc. Matick. MA. USA) (S3 Table). Cumulative growing degree days from veraison to harvest (DD_vh_) were calculated by dividing degree hours higher than 10°C by 24 (S3 Table) [22].

### Vineyard micrometeorological data

Fruit zone temperature of the studied vineyard was measured by temperature loggers (Tinytag transit 2, Gemini Data Logger Ltd, Chichester, UK) in the 2012–13 and 2013–14 growing seasons to validate the estimated temperature data from the STM. In the 2012–13 growing season, one logger (L2013A) was placed in the canopy of a representative vine, while the second logger (L2013B) was placed in another representative vine canopy covered by a commercial UV-stabilised high density polyethylene (HDPE) shade cloth, which blocks 57% of light and 60% of UV (Coolaroo, Gale Pacific Ltd, Australia). In the 2013–14 growing season, one logger (L2014A) was placed in a representative vine canopy, while the second logger (L2014B) was placed in a shading box, and installed next to the latter logger. The shading boxes used in this experiment were similar to those described in Downey et al. [28], which are made from white polypropylene sheeting painted black on the inside. Artificial shadings were used to minimise direct solar radiation to temperature loggers, and therefore shaded loggers could better reflect the air temperature at the bunch zone. The logger has an operational temperature range of -40°C to 70°C with a 0.01°C resolution. Temperature was measured and logged every 30 mins from veraison to harvest (14^th^ Feb—10^th^ Apr 2013 and 8^th^ Feb—8^th^ Apr 2014). The recorded data was downloaded and analysed using the Tinytag Explorer software (version 4.7, Gemini Data Logger Ltd, Chichester, UK) following the method described previously [27].

### Rotundone accumulation model

**Fig 2:**
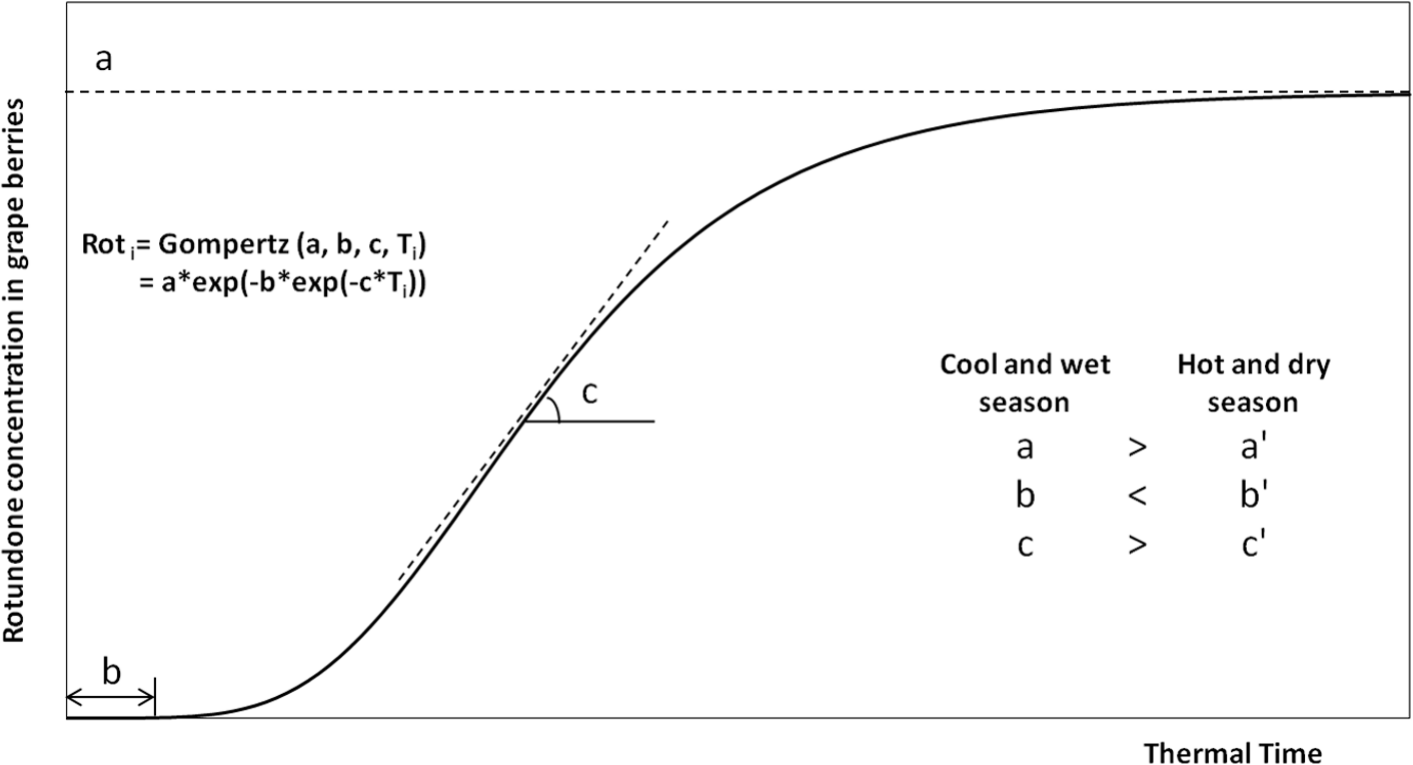
Rotundone accumulation model (RAM) describing the accumulation dynamics of rotundone in grape berries from veraison to harvest using the Gompertz function. In the Gompertz function, Rot_i_ = Gompertz (a, b, c, T_i_), Rot_i_ is the rotundone concentration of grape berries at the time T_i_, parameter a, b and c represents plateau, displacement along time axis and curve growth rate, respectively. Cool and wet seasons are assumed to have higher a, c and lower b compared to hot and dry seasons (a’, b’ and c’).

A rotundone accumulation model was proposed to characterise the accumulation dynamics of rotundone in grape berries after veraison based on the Gompertz function, which was parameterised using MatLab ver. 2014a (The MathWorks, Inc. Matick. MA. USA) as follows:

$${\text{Rot}}_{\text{i}}=\text{Gompertz }\left(\text{a},\text{ b},\text{ c},{\text{ T}}_{\text{i}}\right)\;={\text{ ae}}^{-{\text{be}}^{-{\text{cT}}_{\text{i}}}},$$

where Rot_i_ is the rotundone concentration of grape berries at the time point T_i_ during grape ripening process (Fig 2). The parameter ‘a’ is the upper asymptote, which represents the maximum concentration of rotundone the grape may achieve in a specific season. The parameter ‘b’ sets the displacement of the Gompertz curve along the time (T) axis, and therefore determines when rotundone in grape starts to increase rapidly. The parameter ‘c’ sets the growth rate of the Gompertz curve, which reflects the speed of rotundone accumulation in grape berries.

### Statistical analysis

Estimated temperatures using the STM and observed vineyard temperature data were compared using the CoStat software (version 6.4, CoHort Software, Monterey, USA). Weather data from BOM station, AWAP based weather data, calculated weather data from STM and wine rotundone concentration (Rot_w_) were analysed using principal component analysis (PCA) with a customised code written in the MatLab ver. 2014a (The MathWorks, Inc., Matick, MA. USA). The statistical parameters and significance of relationships between weather parameters and Rot_w_ were calculated using the CoStat software (version 6.4, CoHort Software, Monterey, USA). Weather data and Rot_w_ were also analysed with the k-mean clustering and stepwise linear discriminant analysis (SLDA) using SPSS 21 (SPSS Inc., Chicago, IL. USA).

## Results and Discussions

### Regional weather condition of the studied vineyard

In the past 20 years, the studied wine region experienced a relatively warm period, as 11 out the 15 studied seasons had MJT higher than long-term MJT (18.9°C) (S1 Table) described as climatic condition for the area. This is consistent with a climate variation study showing that annual temperature of major Australian wine growing regions has been increasing in the past 20 years and will continue increasing in the next 40 years [29, 30]. Despite of the observed increments in temperature, the average MJT of all studied seasons (19.8°C) is still within the range of a cool climate wine region classification (MJT<20.9°C) [23]. However, four of the selected seasons (1998–99, 2005–06, 2011–12 and 2013–14) had MJT above this range, and could be considered as warm seasons. Seasons with both high and low E_s_ were selected for this study to investigate the influence of solar exposure on the target quality trait analysed (rotundone). The 1995–95, 2005–06 and 2010–11 growing seasons had low level of E_s_, which was high for the 2006–07 and 2007–08 growing seasons (S1 Table). The studied seasons also had a wide variation of DD_s_ ranging from 1,060 up to 1,424 degree days. Large variation in precipitation was also observed among the studied seasons from a minimum of 124 mm up to a maximum of 605 mm. Vineyard irrigation volume was relatively small compared to the precipitations, which ranged from 0 to 112 mm of water applied per season (Oct-Harvest). ET_c_ did not vary as much as precipitation among the studied seasons, from 328 to 420 millimetres.

### Validation of the interpolated weather data

**Fig 3:**
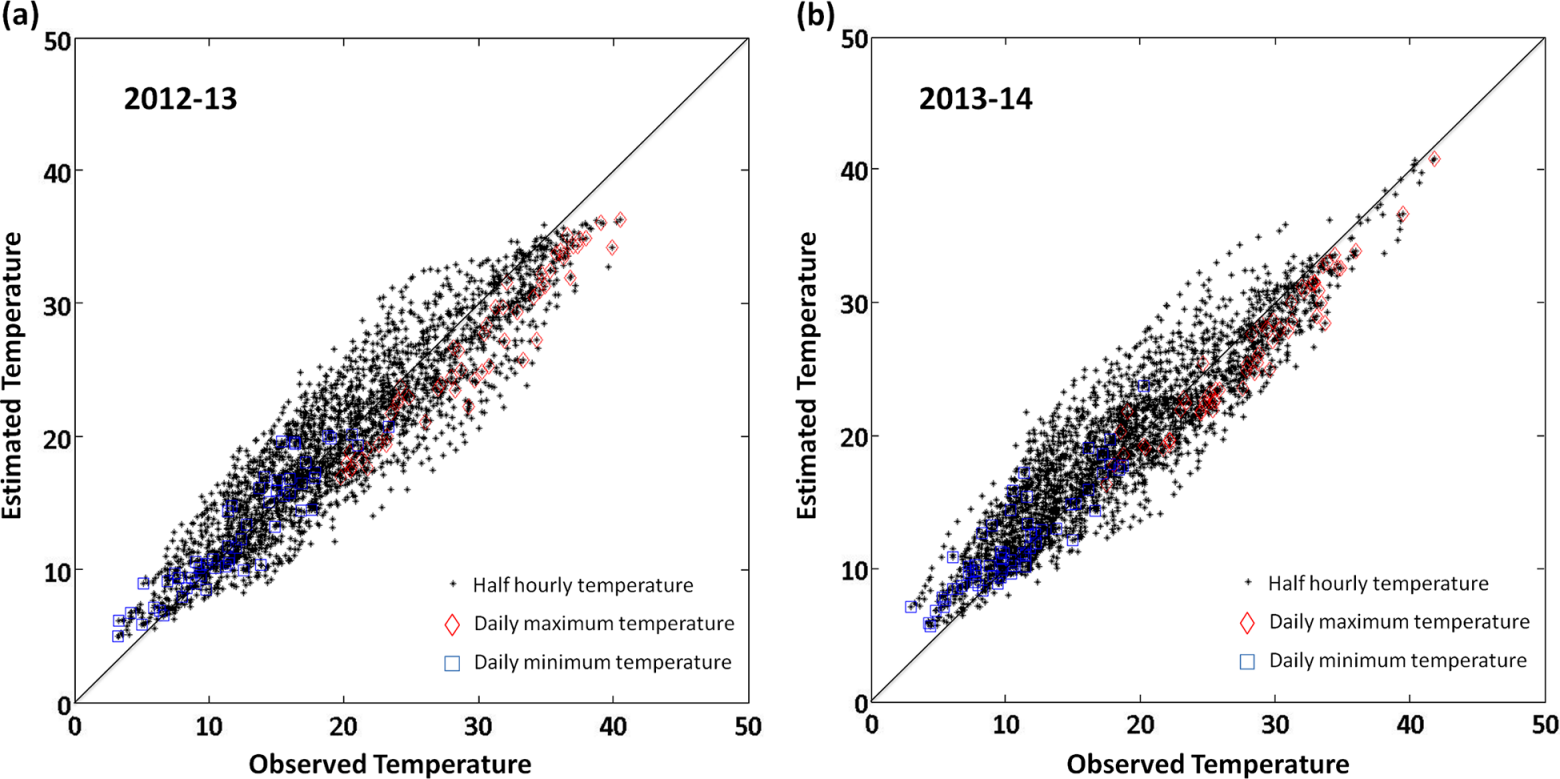
Validation of simulated temperature model. A linear regression is established between observed and estimated temperature for (a) 2012–13 growing season, from temperature data from artificially shaded temperature logger (L2013B), y = 0.8424x+3.383 (R^2^ = 0.84, RMSE = 2.855, *p*<0.0001); (b) 2013–14 growing season, using temperature data from artificially shaded temperature logger (L2014B), y = 0.8139x+4.228 (R^2^ = 0.86, RMSE = 2.478, *p*<0.0001). AWAP daily maximum and minimum temperature used in STM were illustrated in red and blue colour, respectively.

A linear regression analysis was performed between observed (temperature loggers) and estimated data (from AWAP and STM) for both 2012–13 and 2013–14 growing seasons using the curve fitting tool in MatLab (Fig 3). A strong positive linear relationship was established for both 2012–13 (Fig 3a, y = 0.8424x+3.383, R^2^ = 0.84, RMSE = 2.855, *p*<0.0001) and 2013–14 (Fig 3b, y = 0.8139x+4.228, R^2^ = 0.86, RMSE = 2.478, *p*<0.0001) growing seasons. The STM consistently underestimated daily maximum temperature and overestimated daily minimum temperature (Fig 3). Compared to the loggers (L2013A and L2014A) positioned in representative canopies of vines, the predicted percentage of degree hours from artificially shaded loggers (L2013B and L2014B) were closer to the observed value. Artificially shaded loggers (L2013B and L2014B) had minimum influence from direct solar exposure, and therefore their data better reflected the bunch zone air temperature. Thus, the estimated temperature and degree hours from the STM model were suitable to represent the bunch zone air temperature of the studied vineyard.

### Limitations of AWAP dataset and STM estimation

The BOM high-resolution spatial climate maps provides data to accurately estimate vineyard temperatures over time. However, the accuracy of the AWAP weather data can be hampered by two factors: i) the AWAP spatial climatic maps have a resolution of 0.05° (longitude) x 0.05° (latitude) (approximately 5 Km^-2^), and therefore the diurnal temperature data obtained from AWAP maps were averaged values from an area with a wide variation of plant species and vigour [4]. The measured area on the AWAP maps for the studied vineyard includes high vigour eucalyptus forest surrounding vineyards, as a result the AWAP temperature data could underestimate the maximum temperature from the vineyard (Fig 3); ii) Secondly, large spatial variations in topography, slope and soil properties exists in the studied vineyard [11], which is associated to the variation in vineyard vigour [31, 32]. These variations could explain inconsistency in the interpolation of microclimate data within the studied vineyard, resulting in relatively higher vineyard surface temperature in low vigour area during the day [33]. Therefore, the actual vineyard temperature, especially in lower vigour areas may be higher than the calculated value from AWAP. Despite these differences, the eucalyptus forest and vineyard land area of the studied vineyard has limited variation among studied seasons, and the spatial variation of vigour in the vineyard is relatively stable across different seasons [32]. Therefore, differences between AWAP and actual temperature variations are consistent over years, which validate the use of interpolated temperature from AWAP as an alternative to actual time resolved temperature readings for the comparison of vineyard temperature profiles among different growing seasons.

**Fig 4:**
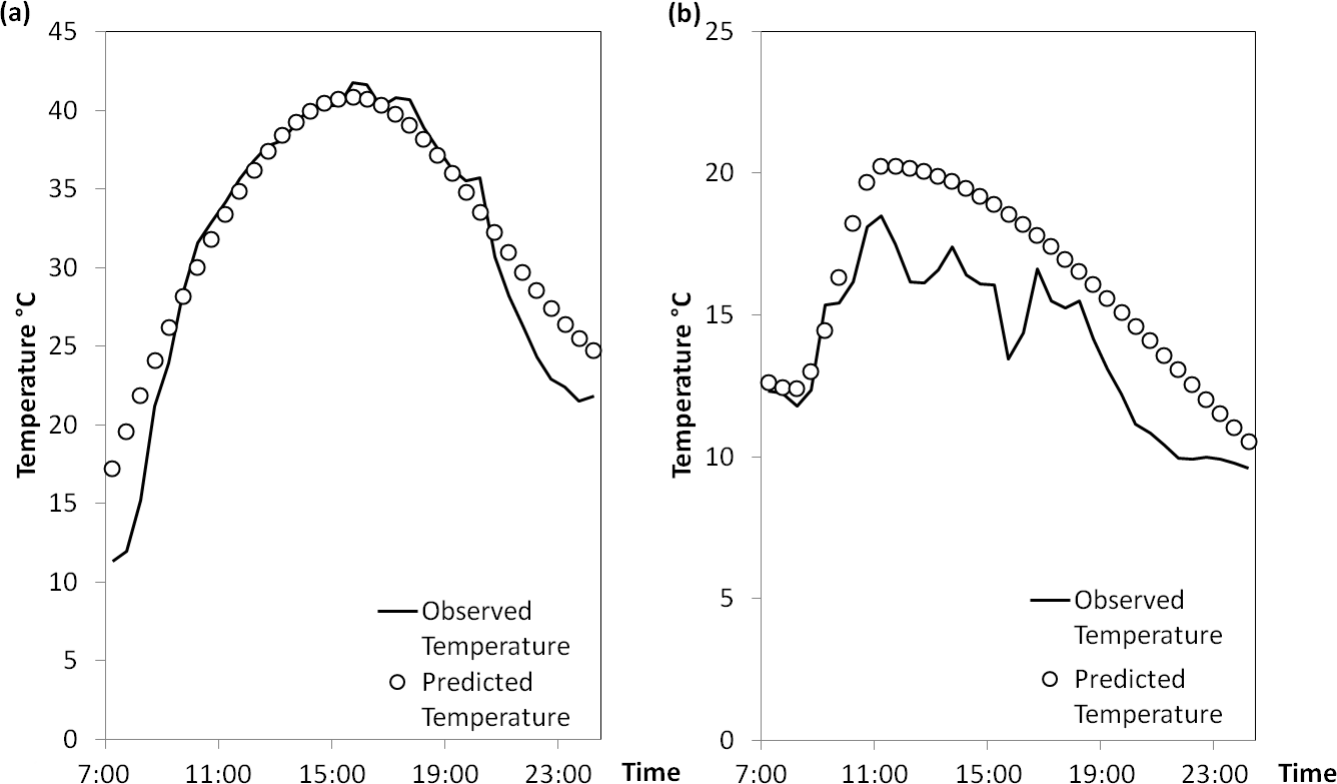
Limitations of the simulated temperature model (STM). (a) Best estimation of half-hourly temperature on a clear day (8^th^ Feb 2014, Day of the year 39). (b) Worst estimation of half-hourly temperature on a partially cloudy day (21^st^ Mar 2014, Day of the year 80).

The STM was established to estimate vineyard bunch zone air temperature over time whenever actual measurements were not available. Even though, the STM described in this paper is site specific, the STM modelling techniques could be applied to most wine growing regions in Australia wherever the high-resolution spatial climate maps data are available. The accuracy of STM largely depends on the temperature data obtained from AWAP, which may underestimate vineyard diurnal temperature (Fig 3). As a result, the predicted percentage of degree hours from STM (S3 Table) is relatively smaller than the observed values (S4 Table). Similarly as the differences between AWAP and actual temperature, the differences between estimated and actual degree hours are also consistent over the years. The accuracy of STM is also affected by the cloud fraction during daytime since the ground surface temperature with vegetation layer is affected by this factor [34]. Therefore, temperature changes rapidly within a day with cloud coverage variability. On clear and totally cloudy days, ground surface temperature usually increase/decrease smoothly (Fig 4a), which can be well predicted by the STM. However, on partially cloudy days, where ground surface temperature changes irregularly due to cloud cover variability, the predicted temperature from STM may not accurately reflect the real temperature changes (Fig 4b).

### Outputs from AWAP maps, STM and rotundone concentration in wine

The studied seasons had clear differences in the majority of weather parameters obtained from AWAP maps. From all of the seasons studied, 2006–07 and 2007–08 were typically hot with associated water deficits and strong solar exposure from veraison to harvest (T_max_: 26.7 and 27.5°C, T_min_: 12.7 and 12.8°C, P_wb_: -234 and -152 mm, E_vh_: 20.7 and 22.8 MJm^-2^) (S2 Table). While the 1995–96, 1998–99 and 2010–11 seasons were typically cooler and wet seasons, with relatively lower solar radiation from veraison to harvest (T_max_: 21.4, 21.6 and 21.0°C, T_min_: 9.0, 7.9 and 10.2°C, P_wb_: 36.1, 28.2 and 251.8 mm, E_vh_: 14.5, 17.0 and 14.2 MJm^-2^) (S2 Table). The 2003–04 growing season was a typically cool season, but with water deficit and moderate solar exposure from veraison to harvest (T_max_: 22.6°C, T_min_: 9.0°C, P_wb_: -104.9 mm, E_vh_: 17.8 MJm^-2^). The remaining seasons had relatively moderate weather parameters (S2 Table) representative of the climate classification for the region. Initial data analysis showed that degree hours rather than mean temperatures were more suitable for modelling the relationship between vineyard temperature and Rot_w_. For example, the 2007–08 growing season only had slightly higher T_max_ (27.5°C), compared to the 2006–07 growing season (26.7°C), while it had much higher DH_30_ (2007–08: 3.47%, 2006–07: 2.46%) (S3 Table). This is indicative of extreme hot weather in the 2007–08 growing season. Cool seasons did not necessarily have consistent low temperature from the veraison to harvest period. Even though the 2003–04 growing season had relatively low T_max_ (22.6°C) and T_min_ (9.0°C) compared to other seasons, it also had relatively high percentage of hot weather condition with almost 3% of DH_30_ throughout the season. The highest concentration of rotundone was found in wines from the 1998–99 growing season (116 ng/L), while the 2005–06, 2006–07, 2007–08 growing seasons had similarly low concentration of rotundone (3.6, 4.6 and 2.5 ng/L; S2 Table). All five growing seasons (1995–96, 1998–99, 2001–02, 2010–11 and 2011–12) with moderate to high concentration of rotundone (60.3, 115.9, 60.5, 52.5 and 67.1 ng/L, respectively) had none or very low DH_35_, and relatively low DH_30_ and DH_25_ (S3 Table).

### Impacts of climatic parameters on wine rotundone concentration (Rot_w_)

From the PCA, it can be seen that the first two principal components combined explained almost 76% of the total variability in the data. The 15 studied seasons were widely spread in the PCA biplots, showing that there were high variation among seasons in weather parameters and Rot_w_. Seasons were separated along PC1 (59%) mainly on the basis of P_wb_, Rot_w_, E_vh_, T_max_, T_min_ and DH_25_. PC1 shows positive correlation between Rot_w_ and P_wb_. An inverse correlation was found for the previous two variables with E_vh_, T_max_, T_min_ and DH_25_. PC2 (16%) separated the seasons mainly according to the MJT, DD_s_ and DD_vh_.

The relationships between Rot_w_ and individual weather parameters were further analysed (Table 1). A positive exponential curve was established between Rot_w_ and P_wb_ (y = 37.84e^0.0028x^, R^2^ = 0.26, RMSE = 29.23, *p* = 0.0095) (Table 1). Wine rotundone was also found to have a significant exponential relationship with T_max_ (y = 117000e^-0.3489x^, R^2^ = 0.51, RMSE = 23.79, *p* = 0.0003), T_min_ (y = 9593e^-0.5780x^, R^2^ = 0.58, RMSE = 22.17, *p* = 0.0385) and E_vh_ (y = 2799e^-0.2610x^, R^2^ = 0.32, RMSE = 28.06, *p* = 0.0003) (Table 1). Rot_w_ was further analysed against calculated percentage of degree hours obtained from the STM, and it was found to have an exponential relationship with DD_vh_ (y = (11410000e^-0.0386x^, R^2^ = 0.53, RMSE = 23.35, *p* = 0.0166), DH_35_ (y = 55.56e^-14.39x^, R^2^ = 0.36, RMSE = 27.15, *p* = 0.0310), DH_30_ (y = 85.96e^-0.7443x^, R^2^ = 0.53, RMSE = 23.43, *p* = 0.0004) and DH_25_ (y = 157.70e^-0.4031x^, R^2^ = 0.61, RMSE = 21.32, *p*<0.0001) (Table 1). No significant correlations could be established between Rot_w_ and other climatic parameters including MJT, E_s_ and DD_s_ (Table 1).

**Table 1:** Exponential relationships (y = a exp(bx)) between rotundone concentration in wine (Rot_w_) and climatic parameters.

Climate factors		Coefficients		SSE^f^	R-square	RMSE^g^	P value
		a	b				
**BOM** ^c^ **data**	**Mean January temperature °C (MJT)**	38.61	-0.0098	15070	0.0001	34.05	0.3742
	**Mean daily solar exposure MJm** ^**-2**^ **(E**_**s**_ **)** ^**a**^	571.2	-0.1350	13960	0.0741	32.76	0.1324
	**Cumulative growing degree days (DD**_**s**_ **)** ^**a**^	6319	-0.0043	11510	0.2364	29.75	0.0805
**AWAP** ^d^ **data**	**Water Balance (P**_**wb**_ **)** ^**a**^	37.84	0.0028	11110	0.2632	29.23	0.0095
	**Mean maximum temperature °C (T**_**max**_ **)** ^**b**^	117000	-0.3489	7356	0.5120	23.79	0.0003
	**Mean minimum temperature °C (T**_**min**_ **)** ^**b**^	9593	-0.5780	6389	0.5761	22.17	0.0385
	**Mean daily solar exposure MJm** ^**-2**^ **(E**_**vh**_ **)** ^**b**^	2799	-0.2610	10240	0.3208	28.06	0.0003
**STM** ^e^ **data**	**Cumulative growing degree days (DD**_**vh**_ **)** ^**b**^	1.141e+07	-0.0386	7091	0.5296	23.35	0.0166
	**% of Degree days above 35°C (DH**_**35**_ **)** ^**b**^	55.56	-14.39	9581	0.3643	27.15	0.0310
	**% of Degree days above 30°C (DH**_**30**_ **)** ^**b**^	85.96	-0.7443	7138	0.5264	23.43	0.0004
	**% of Degree days above 25°C (DH**_**25**_ **)** ^**b**^	157.7	-0.4031	5908	0.6080	21.32	<0.0001

^a^The climate data is for the period from October to harvest.

^b^The climate data is for the period from veraison to harvest of each season. Veraison is approximately 15^th^ February for most seasons. For seasons harvested early than 15^th^ Apr, the approximate veraison time is 60 days before harvest.

**Fig 5:**
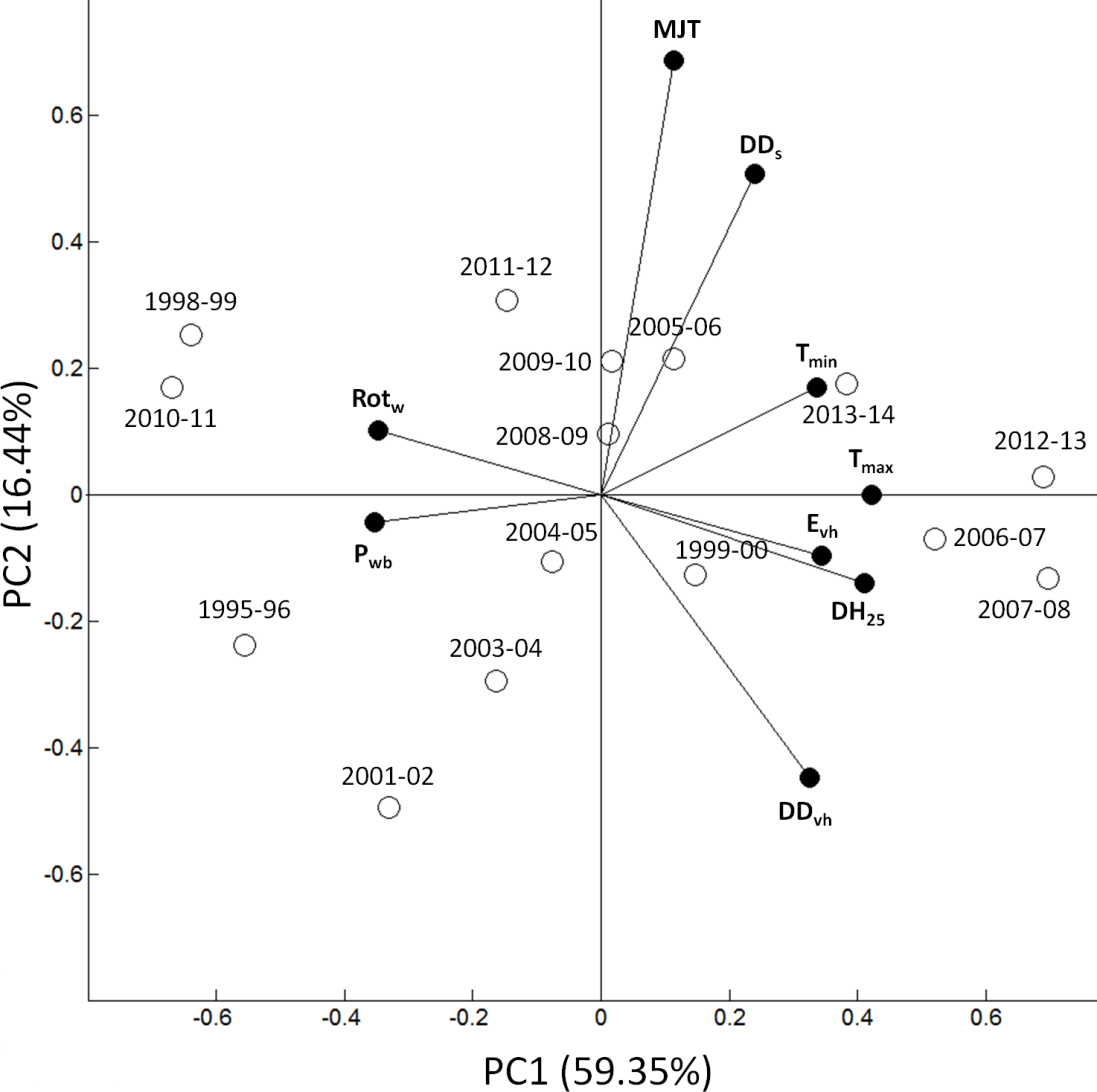
Principal component analysis biplot of the mean PC scores of each weather parameters as vectors, together with the PC scores of each season. PC1 and PC2 count for 59% and 16% of total variance, respectively. The abbreviations used in this figure are wine rotundone concentration (Rot_w_), mean maximum temperature (T_max_), mean minimum temperature (T_min_), mean daily solar exposure (E_vh_), percentage of degree hours >25°C (DH_25_), mean January temperature (MJT), vineyard water balance (P_wb_), cumulative growing degree days from October to harvest (DD_s_) and cumulative growing degree days from veraison to harvest (DD_vh_).

Differences in Rot_w_ between seasons appeared to reflect the combined influence of weather parameters along PC1. T_max_, T_min_, DH_25_ and DD_vh_ represent the temperature condition of vineyard microclimate, while E_vh_ is also associated with grape surface temperature. Negative relationships were observed between wine rotundone and T_max_, T_min_, DH_25_, DD_vh_, E_vh_ (Table 1), thus a higher T_max_, T_min_, DH_25_, DD_vh_, and E_vh_ are likely to result in lower Rot_w_ (Fig 5). This was consistent with a previous study that demonstrated the negative impacts of bunch surface and bunch zone temperature on grape rotundone concentration [12]. Furthermore, sunlight exposure was found to enhance grape monoterpene concentration in Traminette [17], while elevated UV-B radiation would increase monoterpenes and a sesquiterpene, namely E-nerolidol in Malbec leaves [21]. On the contrary, complete exclusion of sunlight was found to inhibit the synthesis and accumulation of monoterpenes and a sesquiterpene, caryophyllene in Muscat [18]. At this stage, no information, as far as the authors’ knowledge, is available about the regulation of sesquiterpene synthesis in *V*. *vinifera* cv. Shiraz by sunlight exposure and ambient temperature. Therefore, further studies are required to separately investigate the influence of direct solar illumination and indirect solar radiation induced temperature increase on grape rotundone concentration.

Water balance was also associated with rotundone concentration as shown by the relationship between P_wb_ and Rot_w_ (Table 1). Thus, a higher overall water balance would lead to increased rotundone concentration in wine (Fig 5), which was consistent with a previous study in *V*. *vinifera* cv. Duras [24]. This effect may have two main reasons: i) increased water availability can lead to higher vine vigour [35], resulting in increased bunch zone shading and lower bunch zone air temperature [33], which tends to promote rotundone production and accumulation in grape berries [12]; ii) higher vigour vines with increased mass of leaves and stems organs may result in increased rotundone from non-grape sources. Higher concentration of rotundone was reported in grape leaves and stems compared to berries [36]. The same study also reported that fermentation with these non-grape materials amongst harvested grapes could lead to elevated rotundone concentration in the resulted wine. In addition, a potential source-sink relationship could exist between leaves/stems and grape berries, as it has been reported that some monoterpene derivatives could be translocated via phloem transportation in other plants [37, 38]. In *V*. *vinifera*, an active transport mechanism may be necessary for translocation of terpene related compounds into grape berries via phloem [39], and this warrants further investigation.

The weather parameters MJT, DD_s_ and E_s_ are commonly used as indices to describe wine region seasonal temperature and solar radiation conditions [23]. However, no significant correlations were observed between Rot_w_ and these parameters (Table 1). This may be due to the development time of rotundone, which mainly accumulates in berries from veraison to harvest in *V*. *vinifera* cv. Duras [24] and Shiraz as described in this paper. This may explain findings that showed rotundone production been sensitive to ambient temperature (T_max_, T_min_, DD_vh_ and DH_25_) and solar exposure (E_vh_) only from veraison to harvest (Table 1). Furthermore, DD_s_ and MJT were found on the opposite side from DD_vh_ along PC2 (Fig 5), which indicated that a warmer overall season might not necessarily indicate a warm post-veraison ripening period, and vice versa. Therefore, traditional seasonal weather indicators used in the viticultural industry (MJT, DD_s_ and E_s_) are not suitable for wine quality studies focused on certain quality traits as rotundone concentration. Further research is required to characterise the influences of environmental factors on the sesquiterpene biosynthesis in *Vitis vinifera* tissues, including rotundone and its precursor α-guaiene.

### Wine rotundone projection based on DH25

**Fig 6:**
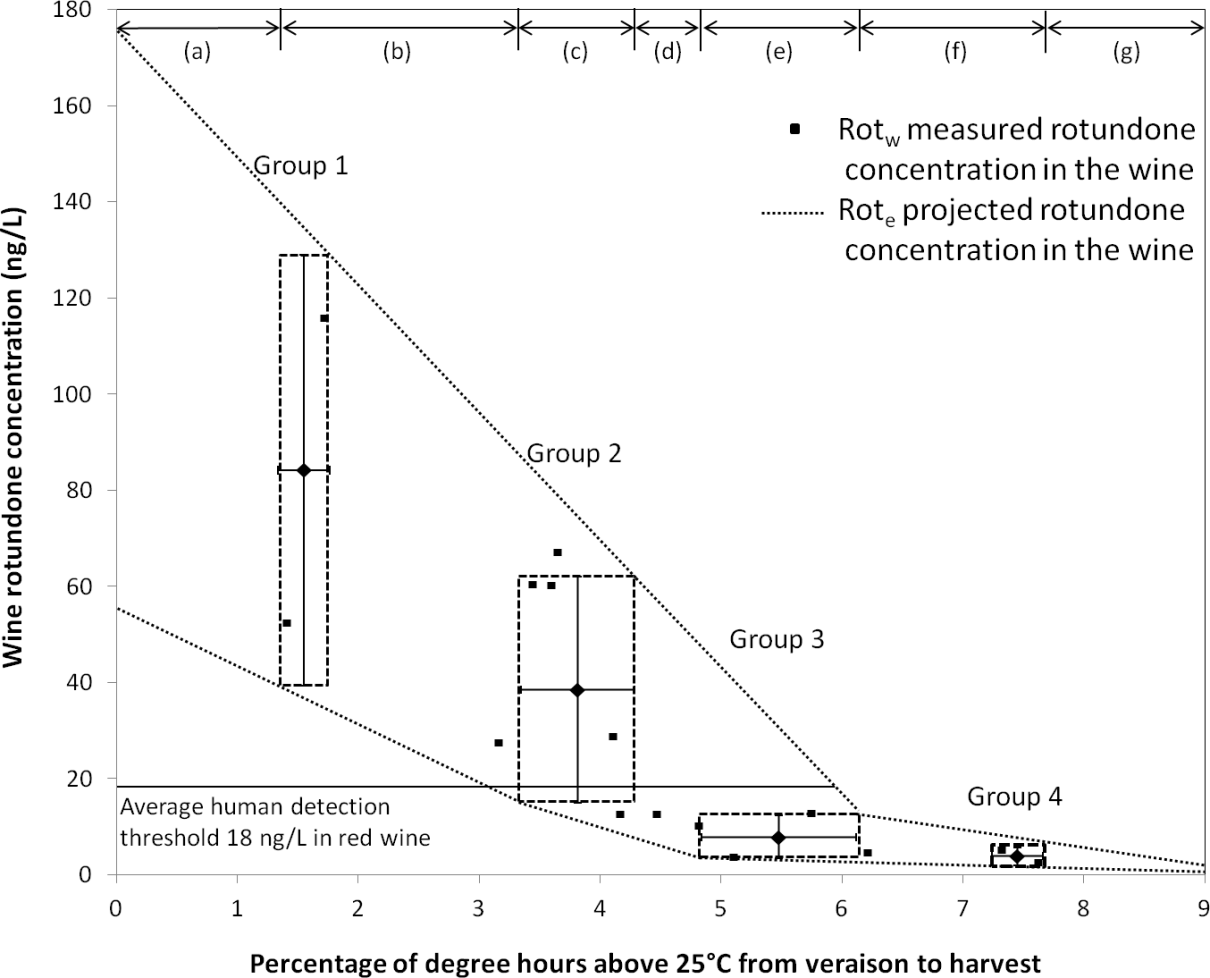
Projection of wine rotundone concentration (Rot_e_) range based on the percentage of degree hours above 25°C (DH_25_) from veraison to harvest. The dot points indicate each studied season. The dash lines connecting each seasonal group indicate the Rot_e_ range at a specific DH_25i_, specified in S6 Table.

For practical applications in the wine industry, it is necessary to use the least number of weather parameters to characterise Rot_w_. From this study and analysing all the weather parameters and their relationships with Rot_w_, the DH_25_ was the most significant vector in separating seasons (Fig 5), and it also had the most significant relationship with Rot_w_ (*p*<0.0001) (Table 1). Using this criterion, the studied seasons were separated into four groups based on K-mean clustering analysis using DH_25_ in SPSS 21 (SPSS Inc., Chicago, IL. USA) (S5 Table). The separated seasonal groups also discriminated according to Rot_w_, T_max_, T_min_, E_vh_, DD_vh_ and P_wb_ (S5 Table). Therefore, DH_25_ could be potentially used as a potential predictor of Rot_w_. This could provide a convenient way for wineries and viticulturists to estimate Rot_w_ from the weather experienced in a specific growing season. The DH_25_ and Rot_w_ range of each k-mean clusters (S5 Table) were plotted with areas demarked between groups by dash lines (Fig 6). A projection of wine rotundone concentration range (Rot_e_) could be generated based on the DH_25_. Thus,

$${\text{Rot}}_{\text{e}}=\;f\;\left({\text{DH}}_{25i}\right),$$

where Rot_e_ is the rotundone concentration range of grape berries at the DH_25_ level of a specific growing season (Fig 6). The dash lines connecting each seasonal group shows the Rot_e_ range at a specific DH_25i_, with two exceptions. If DH_25i_ is lower than the range of group 1 (Fig 6 region a) or higher than the range of group 4 (Fig 6 region h), where no actual wine data is available, Rot_e_ is likely be within the range indicated by the dash lines. The detailed Rot_e_ projection at each DH_25i_ range is specified in S6 Table. Since the averaged human detection threshold for rotundone is 18 ng/L in red wine [7], it is highly unlikely to have detectable rotundone in wine if DH_25i_ is higher than 6%, and most likely to have detectable rotundone in wine if DH_25i_ is lower than 3% (Fig 6). Based on this results and the STM modelling technique proposed in this paper, an Australia wide potential peppery aroma production map could be generated to estimate wine regions capable of producing detectable concentrations of rotundone in Shiraz wine. This may also be related with future climate projections of Australian wine producing regions to estimate the potential peppery wine production regions in the future.

### Seasonal pattern differentiation

**Fig 7:**
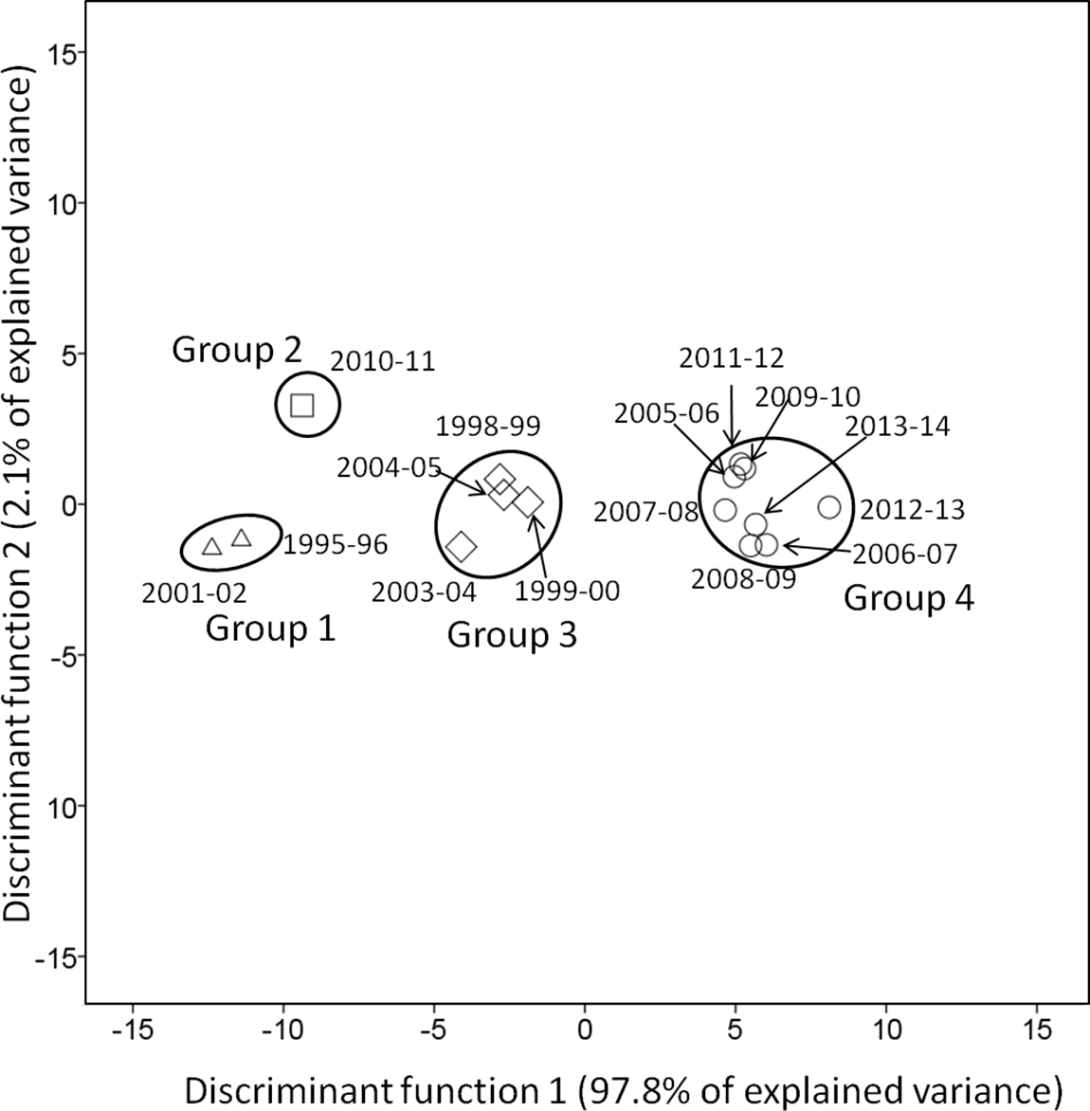
Stepwise linear discriminant analysis (SLDA) biplots illustrating a pattern of similarity in weather parameters between different seasons. Numbers in the biplots represent the seasons for weather parameters and wine rotundone analysis.

By using SLDA biplots (Fig 7), it is possible to recognise the seasonal patterns of weather parameters, which explained most of the variance (99.9%) with the first two discriminant functions. The SLDA analysis considered P_wb_, DD_s_ and MJT as the most significant variables in the stepwise analysis. Seasons were separated into four groups mainly along the first discriminant function (97.8% of explained variance) by P_wb_ on the negative side, and by MJT and DD_s_ on the positive side of the discriminant function 1. Seasons before 2005 (Group 1, 3) were clearly separated from seasons after 2005 (Group 4) considering P_wb_, DD_s_ and MJT as the major determinants (Fig 7) with 2010–11 growing season as an exemption, which corresponded to a wet season (S2 Table). These results showed that the seasonal weather pattern from the studied vineyard is shifting from a wet and cool type to a drier and warmer type in the past two decades. This is consistent with previous studies showing that south-eastern Australian had and would continue having a gradually decrease in precipitations and an increase annual mean temperature [29, 40, 41]. However, no significant differences in Rot_w_ were observed between seasons before (Group 1, 3) and after 2005 (Group 4). The seasons before 2005 may have overall cool and wet weather throughout the vintage (October-Harvest), but not necessarily for the period between veraison to harvest. The later period is more critical to grape rotundone production in *V*. *vinifera* cv. Duras and Shiraz [24]. Australian temperatures have warmed by 0.9°C from 1910 to 2014, and will continue to increase up to 5.1°C by 2090 [40]. The annual temperature of the Grampian wine growing region is estimated to increase for 2 to 2.4°C by 2050 [29, 30]. Despite of the projected annual temperature increase, seasonal temperature may continue fluctuating, resulting in relatively cooler seasons [40]. In addition, south-eastern Australia is affected by El Niño Southern Oscillation, which is associated with the periodical fluctuations in temperature and precipitation [42, 43]. Therefore, relatively wetter and cooler season is expected with the occurrence of this phenomenon, and it is still possible to produce significant rotundone concentrations in those seasons.

### Rotundone accumulation model

The rotundone accumulation model was established to describe the rotundone accumulation dynamics in grape berries within seasons, and to identify the importance of vine phenological stages in the final grape rotundone concentration. By understanding the behaviour of rotundone concentration according to seasonality, it would help grape growers to maximise their grape rotundone concentration using management strategies, such as irrigation scheduling, canopy management or by selecting the optimum harvest time. Rotundone in grape berries mainly accumulates at late stage of ripening and reaches a relatively stable concentration 44 days after mid-veraison in *V*. *vinifera* cv. Duras [24]. The Gompertz function (Rot_i_ = Gompertz (a, b, c, T_i_)) was proposed here to mathematically describe the accumulation curve of rotundone in grape berries from veraison to harvest. This modelling tool was able to reflect the start time and accumulation rate at the maximum rotundone concentration (or plateau) (Fig 2). In cooler and wetter growing seasons, a higher rotundone concentration in grape is expected (Figs 5 and 6), and therefore a higher parameter ‘a’. Cooler and wetter growing seasons may have earlier accumulation starting point and faster rotundone accumulation rate, and therefore a lower parameter ‘b’ and higher parameter ‘c’ is expected (Fig 2).

**Fig 8:**
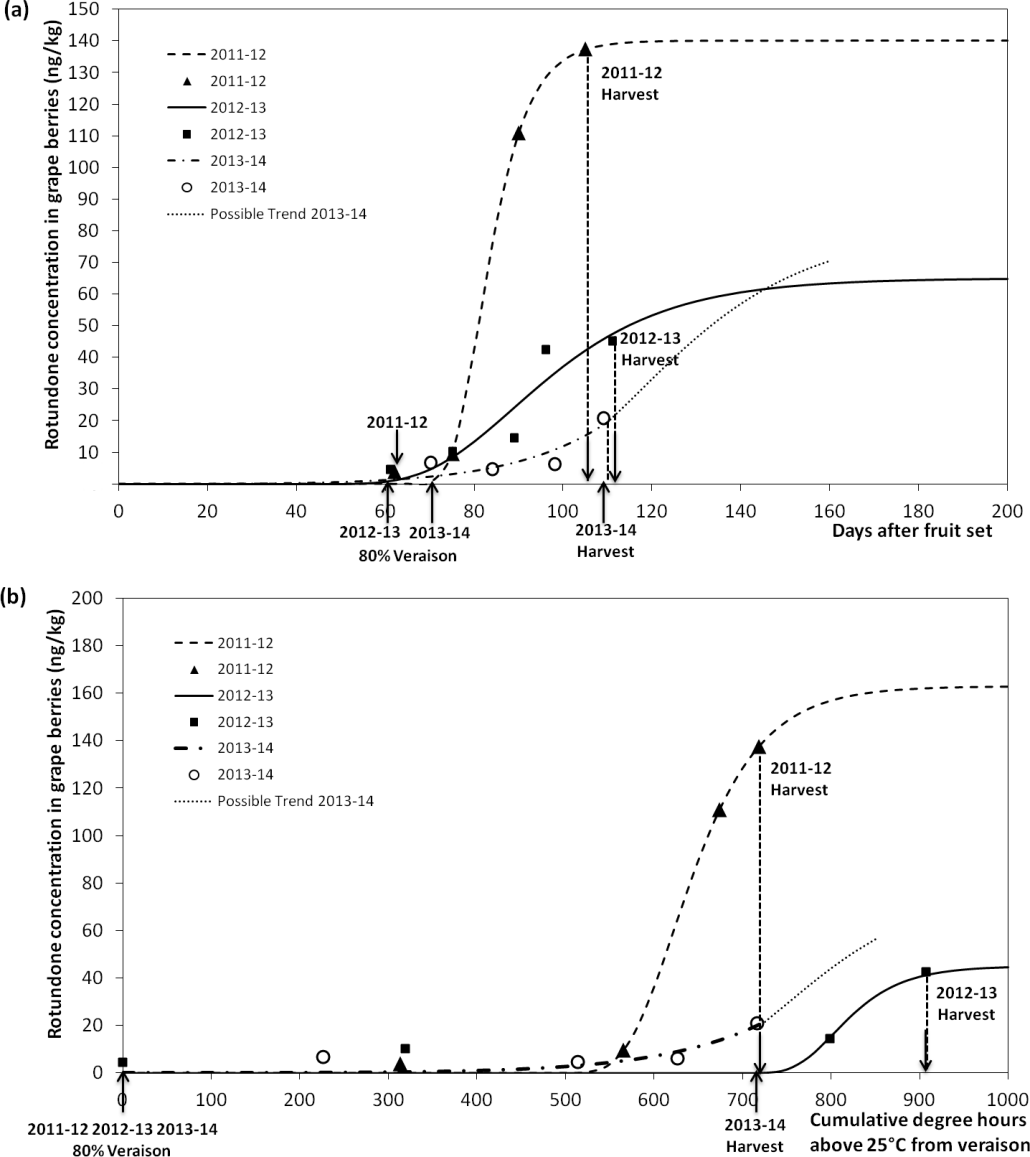
Estimating the accumulation of rotundone in grape berries (Rot_i_) since veraison in three continuous growing seasons using Gompertz function. Estimation based (a) calendar day since fruit set: 2011–12, Rot_i_ = Gompertz (140.2, 522700, 0.1624, T_i_), R^2^ = 0.99, RMSE = 3.74; 2012–13, Rot_i_ = Gompertz (64.96, 99.1, 0.0518, T_i_), R^2^ = 0.85, RMSE = 10.3; 2013–14, Rot_i_ = Gompertz (could not be determined, 17.2, 0.0047, T_i_), R^2^ = 0.73, RMSE = 6.812; based on (b) cumulative degree hours above 25°C from veraison: 2011–12, Rot_i_ = Gompertz (162.8, 95280, 0.01843, T_i_), R^2^ = 0.99, RMSE = 3.73; 2012–13, Rot_i_ = Gompertz (45.09, 124800000, 0.0232, T_i_), R^2^ = 0.90, RMSE = 10.27; 2013–14, Rot_i_ = Gompertz (could not be determined, 20.32, 0.0007036, T_i_), R^2^ = 0.67, RMSE = 7.50.

The Gompertz function accurately described the accumulation trend in rotundone calculated against either calendar days (Fig 8a) (2011–12: Rot_i_ = Gompertz (140.2, 522700, 0.1624, T_i_), R^2^ = 0.99, RMSE = 3.735; 2012–13: Rot_i_ = Gompertz (64.96, 99.1, 0.0518, T_i_), R^2^ = 0.85, RMSE = 10.3), or cumulative degree hours (thermal time) above 25°C (DH_25cum_) (Fig 8b) (2011–12: Rot_i_ = Gompertz (162.8, 95280, 0.01843, T_i_), R^2^ = 0.99, RMSE = 3.73; 2012–13, Rot_i_ = Gompertz (45.09, 124800000, 0.0232, T_i_), R^2^ = 0.90, RMSE = 10.27). However, in the 2013–14 growing season, grape berries samples were harvested before rotundone concentration reached the plateau. As a result, for this season, the Gompertz function only described parameters ‘b’ and ‘c’, but could not predict the plateau parameter ‘a’ (Calendar day: Rot_i_ = Gompertz (could not be determined, 17.2, 0.0047, T_i_), R^2^ = 0.73, RMSE = 6.812; DH_25cum_: Rot_i_ = Gompertz (could not be determined, 20.32, 0.0007036, T_i_), R^2^ = 0.67, RMSE = 7.50). Therefore, at least 44 days from mid-veraison is required before rotundone concentration in berries can reach the plateau for *V*. *vinifera* cv. Duras variety in the Gaillac region (France) (MJT 21.2°C) [24], and it may even take longer for the Shiraz cultivar in a cooler climate region (Grampians, Australia, MJT 18.9°C). In the 2011–12 and 2012–13 growing seasons, the time from 80% veraison to harvest were both longer than 51 days (Fig 8b). Veraison in the 2013–14 growing season occurred around 10 days later than 2011–12 and 2012–13 growing seasons, but was harvested earlier than the other two seasons (Fig 8a). Therefore, a shorter ripening time has resulted in lower rotundone concentration in the 2013–14 growing season. The 2012–13 growing season had clearly higher DH_25cum_, compared to the 2011–12 growing season, which explained it lower maximum rotundone concentration or plateau (Fig 8b). Despite that the 2013–14 growing season had similar DH_25cum_ as the 2011–12 at harvest, the former had much lower rotundone concentration in grape berries. This may by explained by the late veraison time achieved in the 2013–14 growing seasons (Fig 8b). Since the 2013–14 growing season reached a higher concentration of rotundone at a relatively low DH_25cum_ compared to the 2012–13 growing season, it might have reached an even higher maximum rotundone concentration plateau if later harvest date was allowed (Fig 8b). Therefore, vine phenological stage and harvest time selection, especially before rotundone reaches the critical plateau will impact upon the final concentration of rotundone found in grapes and thus wine.

## Conclusions

In this paper, accurate historical weather data was generated from high resolution climate maps and modelled vineyard fruit zone air temperature using the PCHIP modelling approach. Extrapolated data provided a reliable estimation of vineyard temperature in any historical season. The basis of the PCHIP interpolation technique allowed its application to any vineyard located in Australia with similar limited weather information. Fruit zone air temperature, daily solar exposure and vineyard water balance were correlated with the concentration of rotundone in Shiraz wine produced. While the studied vineyard experienced a change in weather conditions after 2005, with an increase in seasonal cumulative GDD and MJT, the rotundone concentration in wine was not affected. MJT, daily solar exposure and GDD over an entire growing season does not relate well to weather indices between veraison to harvest. Temperature from veraison to harvest, in particular the bunch zone air temperature, appeared to represent better the final wine rotundone concentration. Identification of the major environmental factors affecting rotundone concentrations in grape and wine could allow the implementation of precision irrigation techniques and management strategies to manipulate rotundone concentration in grapes at harvest. This study further developed projection models to characterise the possible rotundone concentration range in finished wine, and berry rotundone accumulation trend during the grape ripening process. The use of these two models could allow winemakers to estimate rotundone concentrations in final Shiraz wines based on seasonal climatic conditions, and to adjust winemaking techniques to achieve high quality ‘peppery’ Shiraz wine. This research also provides a guideline to help viticulturists adjust vineyard management practices and identify potentially ‘peppery’ grape growing regions.

## Supporting Information

S1 Table Weather record of the studied wine region in selected growing seasons (Data from Ararat Prison weather station, Australian Bureau of Meteorology Station No. 089085). (DOCX)

S2 Table Summary of weather data for the vineyard in selected growing seasons (Data from Australian Water Availability Project, Australian Bureau of Meteorology) and rotundone concentration in wine (Rot_w_). (DOCX)

S3 Table Summary of thermal data from simulated temperature model from each studied season.^a^
(DOCX)

S4 Table Validation of the simulated temperature model. Regression analysis between predicted temperature data and observed temperature data in growing seasons 2012–13 and 2013–14.
(DOCX)

S5 Table Comparison of the groups separated by k-mean clustering using DH_25_ in wine rotundone concentration and climate parameters. (DOCX)

S6 Table Specification of estimated wine rotundone concentration (Rot_e_) range at different percentage of degree hours above 25°C (DH_25_) from veraison to harvest (Rot_e_ = ƒ (DH_25i_)). (DOCX)
