# Supplementary material for: Environmental Factors and Seasonality Affect the Concentration of Rotundone in Vitis vinifera L. cv. Shiraz Wine
Source: PLoS One. 2015 Jul 15;10(7):e0133137. doi: 10.1371/journal.pone.0133137 (PMC4503395; doi:10.1371/journal.pone.0133137)
Supplement: S1 Table — (DOCX) [file pone.0133137.s001.docx]

**S1 Table. Weather record of the studied wine region in selected growing seasons (Data from Ararat Prison weather station, Australian Bureau of Meteorology Station No. 089085)**

| **Growing Seasons** | **Mean January Temperature °C (MJT)** | **Mean January Maximum Temperature °C** | **Mean January Minimum Temperature °C** | **Mean Daily Solar exposure MJm^-2^(E_s_)^a^** | **Cumulative Growing Degree days (DD_s_) ^a^** | **Total Rainfall millimetres^a^** | **Total Irrigation millimetres^a^** | **Total Evapotranspiration millimetres (ET_c_)^a^** |
| --- | --- | --- | --- | --- | --- | --- | --- | --- |
| **1995-1996** | 18.4 | 25.9 | 10.8 | 19.6 | 1059.9 | 246.2 | 93.3 | 333.3 |
| **1998-1999** | 21.5 | 30.0 | 13.0 | 21.3 | 1188.3 | 263.8 | 112.0 | 362.6 |
| **1999-2000** | 18.7 | 25.9 | 11.4 | 22.0 | 1277.8 | 242.4 | 78.9 | 386.3 |
| **2001-2002** | 17.3 | 25.1 | 9.4 | 20.2 | 1072.3 | 248.2 | 53.3 | 328.3 |
| **2003-2004** | 16.7 | 24.3 | 9.1 | 21.3 | 1244.2 | 201.8 | 46.9 | 382.3 |
| **2004-2005** | 19.1 | 27.0 | 11.1 | 21.4 | 1260.2 | 257.2 | 21.3 | 362.2 |
| **2005-2006** | 21.3 | 29.5 | 13.1 | 18.4 | 1336.7 | 240.0 | 46.9 | 400.0 |
| **2006-2007** | 20.6 | 27.6 | 13.5 | 24.3 | 1268.3 | 163.2 | 28.3 | 420.0 |
| **2007-2008** | 20.6 | 28.4 | 12.8 | 24.1 | 1303.0 | 187.2 | 51.7 | 374.9 |
| **2008-2009** | 20.3 | 29.9 | 10.6 | 22.3 | 1270.0 | 134.8 | 32.0 | 406.0 |
| **2009-2010** | 19.6 | 28.5 | 10.6 | 22.7 | 1424.3 | 296.1 | 25.6 | 404.6 |
| **2010-2011** | 20.3 | 26.9 | 13.6 | 19.3 | 1245.4 | 605.1 | 0.0 | 341.2 |
| **2011-2012** | 21.0 | 28.3 | 13.7 | 21.9 | 1373.3 | 227.8 | 32.0 | 391.3 |
| **2012-2013** | 20.0 | 28.8 | 11.1 | 22.7 | 1392.1 | 124.1 | 82.1 | 415.0 |
| **2013-2014** | 21.7 | 30.0 | 13.4 | 20.8 | 1252.2 | 140.9 | 58.7 | 412.1 |
| **Mean** | 19.8 | 27.7 | 11.8 | 21.5 | 1264.4 | 238.6 | 50.9 | 381.3 |

**^a^**Climate data is for the period from October to harvest of each growing season.
