# Supplementary material for: Environmental Factors and Seasonality Affect the Concentration of Rotundone in Vitis vinifera L. cv. Shiraz Wine
Source: PLoS One. 2015 Jul 15;10(7):e0133137. doi: 10.1371/journal.pone.0133137 (PMC4503395; doi:10.1371/journal.pone.0133137)
Supplement: S2 Table — (DOCX) [file pone.0133137.s002.docx]

**S2 Table. Summary of weather data for the vineyard in selected growing seasons (Data from Australian Water Availability Project, Australian Bureau of Meteorology) and rotundone concentration in wine (Rot_w_)**

| **Growing Seasons** | **Harvest date** | **Mean Maximum Temperature °C (T_max_) ^a^** | **Mean Minimum Temperature °C (T_min_) ^a^** | **Mean Daily Solar exposure**  **MJm^-2^ (E_vh_) ^a^** | **Total Rainfall millimetres ^b^** | **Water Balance millimetres (P_wb_) ^b^** | **Rotundone concentration in wine (ng/L)** **(Rot_w_)** |
| --- | --- | --- | --- | --- | --- | --- | --- |
| **1995-1996** | 10-May-96 | 21.4 | 9.0 | 14.5 | 276.0 | 36.1 | 60.3 |
| **1998-1999** | 5-May-99 | 21.6 | 7.8 | 17.0 | 278.8 | 28.2 | 115.9 |
| **1999-2000** | 13-Apr-00 | 25.8 | 12.0 | 18.1 | 259.6 | -47.7 | 10.2 |
| **2001-2002** | 15-May-02 | 23.1 | 9.1 | 16.3 | 303.3 | 28.3 | 60.5 |
| **2003-2004** | 6-May-04 | 22.6 | 9.0 | 17.8 | 230.5 | -104.9 | 28.8 |
| **2004-2005** | 3-May-05 | 23.3 | 9.4 | 17.8 | 321.5 | -19.3 | 12.6 |
| **2005-2006** | 20-Apr-06 | 24.1 | 10.1 | 18.9 | 260.1 | -93.0 | 3.6 |
| **2006-2007** | 3-Apr-07 | 26.6 | 12.7 | 20.7 | 157.7 | -234.0 | 4.6 |
| **2007-2008** | 21-Mar-08 | 27.5 | 12.8 | 22.8 | 171.6 | -151.6 | 2.5 |
| **2008-2009** | 22-Apr-09 | 23.7 | 10.0 | 16.4 | 145.0 | -229.0 | 12.6 |
| **2009-2010** | 12-Apr-10 | 24.2 | 12.2 | 17.5 | 281.8 | -97.2 | 27.6 |
| **2010-2011** | 5-May-11 | 20.9 | 10.2 | 14.2 | 593.0 | 251.8 | 52.5 |
| **2011-2012** | 18-Apr-12 | 23.9 | 11.0 | 15.9 | 282.20 | -77.1 | 67.1 |
| **2012-2013** | 10-Apr-13 | 27.1 | 18.3 | 18.3 | 126.4 | -206.5 | 5.2 |
| **2013-2014** | 8-Apr-14 | 26.1 | 17.2 | 17.2 | 168.70 | -184.7 | 12.9 |
| **Mean** | na^c^ | 24.1 | 11.4 | 17.6 | 257.08 | -73.4 | 31.8 |

**^a^**The weather data is for the period from veraison to harvest of each season. Veraison is approximately 15^th^ February for most seasons. For seasons harvested early than 15^th^ Apr, the approximate veraison time is 60 days before harvest.

**^b^**Total rainfall and water balance data is for the period from Oct to harvest.

^c^na, not applicable.
