# Supplementary material for: Environmental Factors and Seasonality Affect the Concentration of Rotundone in Vitis vinifera L. cv. Shiraz Wine
Source: PLoS One. 2015 Jul 15;10(7):e0133137. doi: 10.1371/journal.pone.0133137 (PMC4503395; doi:10.1371/journal.pone.0133137)
Supplement: S3 Table — (DOCX) [file pone.0133137.s003.docx]

**S3 Table. Summary of thermal data from simulated temperature model from each studied season^a^**

| **Growing Seasons** | **Total degree hours** | **Cumulative growing degree days (DD_vh_)** | **% of Degree hours above 35°C (DH_35_)** | **% of Degree hours above 30°C (DH_30_)** | **% of Degree hours above 25°C (DH_25_)** |
| --- | --- | --- | --- | --- | --- |
| **1995-1996** | 20732 | 327.4 | 0.00 | 0.75 | 3.60 |
| **1998-1999** | 18620 | 300.1 | 0.00 | 0.33 | 1.72 |
| **1999-2000** | 17993 | 359.4 | 0.49 | 1.46 | 4.82 |
| **2001-2002** | 22995 | 390.6 | 0.00 | 0.91 | 3.44 |
| **2003-2004** | 20500 | 342.6 | 0.45 | 2.59 | 4.11 |
| **2004-2005** | 20223 | 352.0 | 0.10 | 1.87 | 4.17 |
| **2005-2006** | 17668 | 324.0 | 0.02 | 2.10 | 5.11 |
| **2006-2007** | 18715 | 388.5 | 0.68 | 2.46 | 6.22 |
| **2007-2008** | 19180 | 408.7 | 0.44 | 3.47 | 7.63 |
| **2008-2009** | 18168 | 327.9 | 0.01 | 1.11 | 4.47 |
| **2009-2010** | 17317 | 329.7 | 0.00 | 0.88 | 3.16 |
| **2010-2011** | 19814 | 313.3 | 0.00 | 0.00 | 1.41 |
| **2011-2012** | 17691 | 327.0 | 0.04 | 1.33 | 3.65 |
| **2012-2013** | 18983 | 400.5 | 0.18 | 2.40 | 7.32 |
| **2013-2014** | 17958 | 357.8 | 0.31 | 2.39 | 5.75 |
| **Mean** | 19201.23 | 350.0 | 0.18 | 1.60 | 4.44 |

**^a^**All weather data is for the period from veraison to harvest of each season. Veraison is approximately 15^th^ February for most seasons. For seasons harvested early than 15^th^ Apr, the approximate veraison time is 60 days before harvest.
