# Supplementary material for: Environmental Factors and Seasonality Affect the Concentration of Rotundone in Vitis vinifera L. cv. Shiraz Wine
Source: PLoS One. 2015 Jul 15;10(7):e0133137. doi: 10.1371/journal.pone.0133137 (PMC4503395; doi:10.1371/journal.pone.0133137)
Supplement: S4 Table — Regression analysis between predicted temperature data and observed temperature data in growing seasons 2012–13 and 2013–14. (DOCX) [file pone.0133137.s004.docx]

**S4 Table. Validation of the simulated temperature model. Regression analysis between predicted temperature data and observed temperature data in growing seasons 2012-13 and 2013-14.**

| **Growing Seasons** | **Temperature Loggers** | **Linear Regression Analysis** | | | | | | **Data Type** | **Total degree hours** | **% of Degree hours above 35°C (DH_35_)** | **% of Degree hours above 30°C (DH_30_)** | **% of Degree hours above 25°C (DH_25_)** |
| --- | --- | --- | --- | --- | --- | --- | --- | --- | --- | --- | --- | --- |
|  |  | **Equation** | **R-square** | **SSE**^c^ | **RMSE^d^** | **% of Outliers** | **P value** |  |  |  |  |  |
| 2013 | L2013A **^a^** | y=0.7531x+4.631 | 0.7789 | 29730 | 3.362 | 0.38 | <0.0001 | Ob. Val.^e^ | 17949 | 5.04 | 6.94 | 12.88 |
|  | L2013B **^a^** | y=0.8423x+3.383 | 0.8403 | 21440 | 2.855 | 0.34 | <0.0001 | Ob. Val. | 17328 | 1.39 | 4.93 | 9.90 |
| 2014 | L2014A **^b^** | y=0.7814+4.485 | 0.8528 | 17970 | 2.52 | 0.25 | <0.0001 | Ob. Val. | 17494 | 3.13 | 5.06 | 9.00 |
|  | L2014b **^b^** | y=0.8139+4.228 | 0.8576 | 17360 | 2.478 | 0.28 | <0.0001 | Ob. Val. | 17066 | 0.38 | 3.58 | 8.73 |

^a^Temperature logger L2013A and L2013B measure vineyard temperature in 2013, with L2013A placed inside vine canopy and L2013B placed inside canopy of vine covered with UV-stabilized HDPE shade cloth;

^b^Temperature loggers L2014A and L2014A measure vineyard temperature in 2014, with L2014A placed inside vine canopy and L2014B placed inside polypropylene shade cloth, and then installed next to L2014A.

^c^error sum of square.

^d^root mean square deviation.

^e^observed value.
