# Supplementary material for: Environmental Factors and Seasonality Affect the Concentration of Rotundone in Vitis vinifera L. cv. Shiraz Wine
Source: PLoS One. 2015 Jul 15;10(7):e0133137. doi: 10.1371/journal.pone.0133137 (PMC4503395; doi:10.1371/journal.pone.0133137)
Supplement: S5 Table — (DOCX) [file pone.0133137.s005.docx]

**S5 Table. Comparison of the groups separated by k-mean clustering using DH_25_ in wine rotundone concentration and climate parameters.**

|  | **Group 1** | **Group 2** | **Group 3** | **Group 4** |
| --- | --- | --- | --- | --- |
| **Mean January temperature °C (MJT)** | 20.9±0.8 | 18.9±1.6 | 20.6±1.3 | 20.3±0.4 |
| **Cumulative growing degree days (DD_s_) ^a^** | 1216.9±40.4 | 1243.5±137.6 | 1283.8±36.8 | 1347.6±63.0 |
| **Mean maximum temperature °C (T_max_)^b^** | 21.3±0.5 | 23.2±0.9 | 25.7±1.1 | 27.3±0.3 |
| **Mean minimum temperature °C (T_min_)^b^** | 9.0±1.7 | 10.0±1.2 | 13.0±3.0 | 15.6±3.9 |
| **Water Balance (P_wb_)^a^** | 140.0±158.1 | 66.2±91.9 | 139.9±84.8 | 179.1±38.8 |
| **Mean daily solar exposure MJm^-2^ (E_vh_)^b^** | 15.6±2.0 | 16.6±1.2 | 18.7±1.5 | 20.3±3.2 |
| **Rotundone concentration in wine (ng/L)** **(Rot_w_)** | 84.2+44.8 | 38.5±23.6 | 7.8±4.5 | 3.9±1.9 |
| **Cumulative growing degree days (DD_vh_)^b^** | 306.7±9.3 | 342.5±23.3 | 357.4±26.4 | 404.6±5.8 |
| **% of Degree days above 25°C (DH_25_)^b^** | 1.6±0.2 | 3.8±0.5 | 5.5±0.6 | 7.5±0.2 |

^a^The climate data is for the period from October to harvest.

^b^The climate data is for the period from veraison to harvest of each season. Veraison is approximately 15^th^ February for most seasons. For seasons harvested early than 15^th^ Apr, the approximate veraison time is 60 days before harvest.
