# Supplementary material for: Environmental Factors and Seasonality Affect the Concentration of Rotundone in Vitis vinifera L. cv. Shiraz Wine
Source: PLoS One. 2015 Jul 15;10(7):e0133137. doi: 10.1371/journal.pone.0133137 (PMC4503395; doi:10.1371/journal.pone.0133137)
Supplement: S6 Table — (DOCX) [file pone.0133137.s006.docx]

**S6 Table. Specification of estimated wine rotundone concentration (Rot_e_) range at different percentage of degree hours above 25°C (DH_25_) from veraison to harvest (Rot_e_ =ƒ (DH_25i_)).**

|  | **DH_25i_ range** | **Rot_e_ range** |
| --- | --- | --- |
| **(a)** | DH_25i_ < 1.3% | (-12.16 x DH_25i_ + 55.64) ng/L ≤ Rot_e_ ≤ (-26.58 x DH_25i_ + 175.87) ng/L |
| **(b)** | 1.3% ≤ DH_25i_ < 3.3% | (-12.16 x DH_25i_ + 55.64) ng/L ≤ Rot_e_ ≤ (-26.58 x DH_25i_ + 175.87) ng/L |
| **(c)** | 3.3% ≤ DH_25i_ < 4.3% | (-7.77 x DH_25i_ +40.96) ng/L ≤ Rot_e_ ≤ (-26.58 x DH_25i_ + 175.87) ng/L |
| **(d)** | 4.3% ≤ DH_25i_ < 4.8% | (-7.77 x DH_25i_ +40.96) ng/L ≤ Rot_e_ ≤ (-27.15 x DH_25i_ + 178.31) ng/L |
| **(e)** | 4.8% ≤ DH_25i_ < 6.1% | (-0.59 x DH_25i_ +6.23) ng/L ≤ Rot_e_ ≤ (-27.15 x DH_25i_ + 178.31) ng/L |
| **(f)** | 6.1% ≤ DH_25i_ < 7.7% | (-0.59 x DH_25i_ +6.23) ng/L ≤ Rot_e_ ≤ (-4.19 x DH_25i_ +37.91) ng/L |
| **(g)** | DH_25i_ ≥ 7.7% | (-0.59 x DH_25i_ +6.23) ng/L ≤ Rot_e_ ≤ (-4.19 x DH_25i_ +37.91) ng/L |
